# Supplementary material for: Environmental influences on evolvable robots
Source: PLoS One. 2020 May 29;15(5):e0233848. doi: 10.1371/journal.pone.0233848 (PMC7259730; doi:10.1371/journal.pone.0233848)
Supplement: S1 Appendix — (PDF) [file pone.0233848.s002.pdf]

1 S2 Appendix: extra experiments

1.1 Flat environment versus Lava environment

We carried out two types of experiments using the same experimental setup, except for the environments in which the robots were evolved. In both experiments, the environmental conditions were not seasonal, but static.

The experiments were a) Flat: this is our baseline, and the environment is a plane flat floor; b) Lava: the environment is also a plane flat floor, but this floor is hot. This name is an allusion to "The floor is lava" game. The environments are depicted in Fig 1.

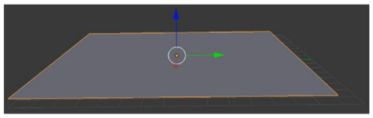

Figure 1: Illustration of the Flat environment. Note that the Lava environment is not shown because structurally it is the same as Flat.

In the Flat environment, the fitness function was defined by Eq. (1):

$$f_1 = \begin{cases} s_x & \text{if } s_x > 0 \\ \frac{s_x}{10} & \text{if } s_x < 0 \\ -0.1 & \text{if } s_x = 0, \end{cases} \tag{1}$$

where s_x is the speed of the robot. The duration t of the evaluation periods was set to 50 seconds in all experiments.

As for the Lava environment, a penalty was added to the fitness function, so to simulate a hot floor, i.e., robots that avoid touching the floor have more chances to succeed. The penalized fitness function is defined by Eq. (2):

$$f_2 = \begin{cases} \frac{f_1}{C} & \text{if } f_1 >= 0 \\ f_1 * C & \text{if } f_1 < 0, \end{cases} \tag{2}$$

where f_1 is the non-penalized fitness function defined by Eq. (1) and C is the penalty representing the average points of contacts of the morphology with the ground, defined by Eq. (3):

$$C = \frac{c_m}{S}, \tag{3}$$

where c_m is the total number of times the modules of the morphology were in contact with the floor during life-time, and S is the total number of modules in the morphology. Each module can have from zero to four points of contact with the floor in each instant of time. Moreover, the reason for averaging the total amount of points of contact relative to the size of the morphology was to depress evolution to exploit tiny robots, i.e., composed of as few modules as possible.

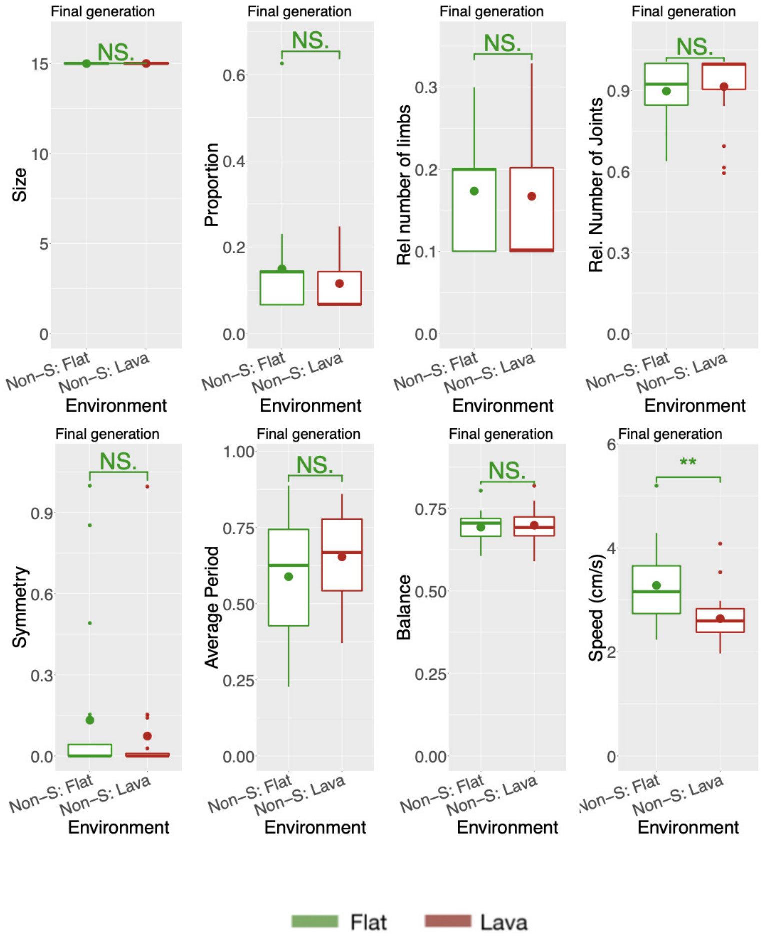

Figure 2: Comparison of morphological and behavioral properties in different environmental conditions. Line plots show the progression of the mean of the population (quartiles over all runs), while boxplots show the mean of the population in the final generation. Significance levels for the Wilcoxon tests in the boxplots are $* < 0.05$, $** < 0.01$, $* * * < 0.001$.

The plots in Fig. 2 show, for the morphological descriptors and behavioral descriptors, the comparison of their average in the final generation. These charts show that the predominant morphological properties of the population of robots evolved in the Lava environment are not different from the ones evolved in the Flat environment. The same can be said for the emergent behavior Balance. After all, the only observable difference concerns the descriptor of speed, which is just the performance of the task itself.
