# Supplementary material for: Environmental influences on evolvable robots
Source: PLoS One. 2020 May 29;15(5):e0233848. doi: 10.1371/journal.pone.0233848 (PMC7259730; doi:10.1371/journal.pone.0233848)
Supplement: Attachment — Submitted filename: Response to Reviewers.pdf [file pone.0233848.s004.pdf]

Thank you so much for all the feedback, which helped us to improve the paper very much. Our answers follow in green in this letter. Furthermore, in the manuscript all changes applied are automatically indicated through latexdiff.

Reviewer #1: This work is a follow up work to [15], where the authors proposed a system based on L-Systems to evolve morphology + neural controllers for locomotion. In [15], they show that their approach can lead to diverse morphology when the environment is changed (i.e. tilting), but in some environments (i.e. obstacles) doesn't really lead to different robots. The main contributions in this work over [15] is addition of the Lava environment, and work on stimulating more diverse morphology.While I really enjoyed reaching [15], honestly I believe this is an incremental work to their earlier work published at GECCO2019 [15], which is a solid paper, and a well-received work at the conference. It is my first time reviewing for PLOS so I'm not sure how it is like, but if this work were to be published at GECCO2020, and I was a reviewer, it would not have enough novelty. But sometimes it is okay for journals to published a polished version of conference papers with minor novelty contributions, so I think in that case it is okay to publish, so this I would like to leave it to the editors to decide. For this reason, I put in "major revision required" but if the editor is fine with publishing a polished version of a fantastic conference paper, then it should be "accept, or minor revision".If more novelty is needed, I have a few ideas that might help make the work better, which moves in the direction of having less hand-engineering, and more ALIFE style emergent behaviors, which should lead to more interesting and diverse results:- Rather than hand-engineering the reward function, think about using one based on survival, and looking at performance of the agents that "survived"? (i.e. not fall over or melt in lava) - I noticed the sinusoidal component is hand-engineered into a locomotion, which feels unsatisfying given that walking with a cyclical motion can be an emergent skill (if sinusoidal gate is part of the search space of the neural controller), rather than hand-engineered feature. - Perhaps thinking about having a population interact with each other in a single environment fighting for survival, and see what behaviors and morphologies occur in "self-play" or "survival of fittest" scenarios. - the environments are not super interesting, to be honest. how about adding rewards like food, giving agents some perception (can be pixel perception of the env, or just distance and direction to nearby items), and see if the agents make use of their sensory inputs, to make things more life like?
[15]https://www.cs.vu.nl/~gusz/papers/2019-Effects_of_environmental_conditions_on_evolved_robot_morphologies_and_behavior.pdf

We agree with the reviewer that adding the Lava experiment/analysis was borderline significant. Therefore, we decided to focus on the tiltable floor environment, while adding new possible scenarios. Specifically, we consider dynamically changing the environmental conditions during the robots' lifetime. Thus, we conducted new experiments that answer a new research question. These experiments are introduced and discussed in sections 4.2 and 5.2 respectively.

Reviewer #2:

Overall, the work does provide promise for future evolutionary robotics. The methodology is sound and does collect substantial data from previous work. However, the broader implications of the experiments seem limited despite the amount of work that is referred to in the paper. Specifically, there are three main points that need attention and require revisions.

First, a clearly stated research objective (whole point of the research – i.e. why these experiments were done at all) and contribution of the results to the state of the art in evolutionary robotics (introduction) and referral to the objective and results significance in the discussion and conclusion sections.

All right, we reformulated the introduction and conclusion making the objectives clearer.

Added to intro: "The main contributions of this work are 1) explicitly putting the issue of the effect of the environment on the Evolutionary Robotics research agenda and noting that it is hard to find different environments that lead to different robots, 2) providing an open source simulator and test suite to facilitate further research, 3) new insights into environmental influences on evolved robot traits. Let us note that it is hard to investigate environmental influences experimentally because it is very difficult to design environments that do induce phenotypic differentiation. That is to say, it is hard to design diverse environments that lead to diverse phenotypes. Notably, while we have experimented with multiple (types of) environments, the cases we present here are the only ones that led to differentiation. Curiously, in these (unpublished) experiments the same type of robots evolved in very different environments. We hypothesize that it has to do with the relative simplicity of these environments compared to the richness of (environmental) factors that determine the selection probabilities in Nature."

Please consult the paper to see the changes applied to the conclusions section.

Second, some discussion of environmental versus evolved morphological complexity is needed (preferably with reference to complexity measures of the environment and creature morphology), and the relationship of this papers results to that of the work presented in [16].

We do agree with the referee that measuring environmental, morphological, and phenotypic complexity is a very interesting and important research goal. However, thanks to this comment we have realized that our poor choice of wording, especially those used in the most general claims in the introduction and discussion sections, have created a misunderstanding about the research contribution of this manuscript. When using the word complex we actually were referring to the richness of what is available in nature, more specifically from the point of view of diversity. We have therefore now toned down these paragraphs, by avoiding the word "complexity" and using the word "differentiation" or "diversity". We now touch and discuss the matter of complexity in the future work description.

Third, an analysis and discussion of the reasons why results differed for the experiments presented in this paper versus those presented in the authors previous work ([15]).

Thank you for spotting this. We now explained it better in the manuscript. The added text reads: A possible reason for this case of~\cite{miras2019effects} is that the task was too difficult. This might have led the search to a local optimum that did not induce the expected phenotypic differences while robots performed poorly on the task. This could indicate limitations in the encoding method, evolutionary algorithm or fitness function.

Each of these points (together with suggestions for minor revisions) are elaborated upon in the following. However, a significant re-write of the introduction, discussion and conclusions sections are needed before this paper can be accepted for publication in this journal.

General comments and corrections:

Remove figure 1 – such a standard evolutionary process is well known, will be familiar to most readers – and is not necessary to reproduce here – rather simply refer to Eiben and Smith (2003).

Done, thank you.

Do not begin new sections so close to the end of page – e.g. section 3.2 and 3.5.

Done, thank you.

Be sure all figures are placed at the top of each page – e.g. figure 3, 4, 5 and 6.

Done, thank you.

Place links to all videos and ancillary material in footnotes.

Done, thank you.

Do not use "/" as a joiner for two words – e.g. "task/environment" – rather say: "task and environment, or both".

Done, thank you.

The function of periods in oscillators in section 4.2.3 needs to be clarified – this section should be expanded a bit for a clearer explanation – as from the example in figure 12 – it is unclear what the oscillators are and what function the Period parameter has in relation.

Done, thank you.

A wider range of test environments (with an information-theoretic measure of environmental complexity – e.g. from low to high complexity environments) will be needed if they wish to make this research at all relevant to the evolution of morphological versus environmental complexity in nature (as the authors claim in the conclusions).

Results and Discussion.

With respect to the following – a measure of environmental complexity would greatly assist in classifying gradations of environments – as well as comparatively (evolved) morphological complexity – and thus give more weight to the results discussion (and conclusions drawn from this discussion).

“Through an initial intuition, we considered that the challenge of the task in the Tilted environment is greater than in the Flat environment, because its whole ground is (gradually) elevated, requiring a robot to climb it. Following this initial intuition, the level of difficulty of the locomotion task in Tilted is indeed higher than in Flat, once robots presented significantly lower speed (Fig 18) in the former.”

All right, added to future work.

Conclusion

“dependence from the environment” --> “dependence on the environment”

Done, thank you.

The following can be removed – rather simply say that observing natural evolution in controlled biological systems is usually not feasible due to the extended time periods of observing N generations and the simple nature (and thus limited morphological versus environment insights) of organisms with short generational periods.

“On the one hand, the natural evolutionary process is too slow to be experienced in a lab, and for most species, no human could experience a significant evolutionary period in her life span. Because of that, evolutionary experimentation is often done with a few specific species that have a short life cycle and rapid evolution, for instance the fruit fly Drosophila subobscura [37], limiting the scope of what can be investigated. On the other hand, artificial evolution is much faster, i.e., experiments can be run in days or weeks.”

Done, thank you.

Also, the following is rather obvious and can be removed:

“Importantly, when we aim at achieving levels of complexity like the ones we observe in nature, it might be essential to reproduce, at least some of, the conditions which allowed nature to achieve these levels.”

Done, thank you.

Despite the following being mentioned a few times – there was no discussion or analysis as to why this paper yielded different results in the evolved behavior-morphology couplings.

“The paper [15] demonstrated an example of a drastic environmental change that did not induce any significant changes neither on morphology nor on behavior.”

Done, thank you!

Introduction

The research goal is too vague and non-specific as to what is being evaluated and what the potential overall contributions of such experimental evaluations.

Done, thank you.

Most importantly – the reason why the authors are doing these experiments at all is missing – in the conclusions it is stated that the role of evolutionary robotics (and assumedly experiments such as these) is to elucidate mechanisms of evolution leading to varied morphological complexities across environments) – however the possibility to explain anything about natural evolution from the results presented is way too tenuous. That leaves us with creating new designs for utilitarian robots to solve various tasks across various environments (also mentioned in the conclusion). However, this is not stated as a goal in the introduction – and the test environments are too far removed from any real-world environment (and the task is simply gait-control) – so that leaves the reader wondering what the actual objective (and thus contribution) of the experiments is exactly.

Done, thank you!

In the current paper, we extend our previous work [15], and our main goal is to gain deeper insights into the effect of the environment on a robotic evolutionary process. Our experiments demonstrate and discuss the effects that diverse environmental conditions can have on behavioral and phenotypic properties of evolvable populations of robots. To this end, we follow an experimental approach. In summary, we consider three different environments, define quantitative descriptors of behavioral, morphological, and controller properties, and compare how they depend on the environments."

The broader implications of the results presented in figure 13 need to discussed a bit more and the contributions of such results clearly stated.

"To better illustrate the differences between the properties of the final populations we plotted density maps with three example pairs of descriptors in Fig 13, allowing a multidimensional perspective. These charts show that the fittest robots evolved in Lava spread to different areas of the space than those that evolved in Flat."

All right, added to the text: Note that robots in Tilted have a higher Balance, a higher Rel. Number of Limbs, and a higher Proportion, demonstrating that it is hard to maintain a stable gait for climbing a hill when a robot has a single limb or when it is disproportionate. Robots in Flat instead do not possess many limbs, are disproportionate, and have a low Balance, because stability is less necessary to locomote on a plane flat floor.

Also, further insights into the relationship between the environment and morphological evolution could be gained from changing the environment type mid-way through evolution, so as (for example) to elucidate the evolutionary conditions for generalists (morphological-function over multiple niches) versus specialists (morphological-function in a specific niche).

The reviewer is right indeed. And changing the environment mid-way through out evolution is what we presented in a previous paper [Miras K, Eiben A. The impact of environmental history on evolved robot properties. (ALife19)]. We now clarify this better in the manuscript. In this revision, we decided to increase the novelty of the paper and we significantly extended our contribution, on the lines proposed by the reviewer, by adding a new setup where we change the environment mid-way through out lifetime. These experiments are introduced and discussed in sections 4.2 and 5.2 respectively.

Also, testing the robustness of evolved coupled behavior, with respect to slightly deformed morphologies across a range of environments could also potentially yield more useful insights about the relationship between evolved behavior-morphologies and the environment. However, this would need to be the subject of more extensive experiments – which do not necessary have to be implemented for this paper's revisions.

The reviewer is right again. This was the subject of previous work. We have now rephrased the manuscript to explain better this past contribution. [Miras K, Eiben A. The impact of environmental history on evolved robot properties. (ALife19)].

Furthermore, while the importance of complexity is mentioned in the abstract and introduction, as well as previous work reviewed ([16]), and the authors seem to be testing the impact of increasingly complex environments on morphological evolution – there is no mention (or measure) of evolved morphological complexity – there was the Relative Number of Limbs metric (which could be construed as some form of complexity) – but results did not seem to present any significant difference for this metric (section 5.1).

At any rate, there needs to be some discussion about evolved morphological complexity relative to environmental complexity and the relationship of the results presented in this article to previous work ([16]) – this is especially important as this is the only other major work that varies the environment to test varying morphological evolution.

All right, added to future work.

Other corrections:

Page Line

16 434-436

Text does not match figure

Text states that "In Fig 15 we see that the average period in the alternative environment (Lava) is significantly higher than in the baseline environment (Flat)." However, the figure shows the difference between environments as not significant. It appears the wrong figure may have been referenced.

You are right, we must have made confusion.

Page Line

2 33-36

Imprecise text. It is not clear why the research in [12,13,14] does not constitute “substantial experimental evidence”.

They do. We meant that these are only a few examples. We clarified it in the text.

3 66-69

Imprecise text. It may be better to state clearly that the environment did not change from a flat plane in [25].

Done, thank you.

3 72-75

Imprecise text. It seems worth mentioning that there was evidence of differences in morphological traits (legged locomotion), but these were not measured or investigated in depth.

Done, thank you.

3 90

Unexplained terminology. The use of the term “modules” is unclear without the context of the later chapters in this paper which describe what "modules" are.

Done, thank you.

3 90

Unclear description. The description of the shape of the robot is difficult to understand without the context of the later chapters in this paper which describe the robot modules.

Done, thank you.

3 95

Imprecise text

It is unclear whether “task/environment” means task “task and environment”, “task or environment”, or “task and/or environment”

Done, thank you.

3 110

Correction “from” should be “for”

Done, thank you.

4 143

Unexpanded acronym. First use of the acronym CPG has not been expanded to full words.

Done, thank you.

5 Eq. 1

Require more explanation. It would be useful to the reader to know what informed the form of this modified sine wave.

Done, thank you.

9 257

Correction “wights” should be “weights”

Done, thank you.

9 260-261

Correction “id” should be capitalised as “ID”

Done, thank you.

9 Fig 4.

Correction Text in arrows should be “move_ref_I(1,1)” instead of “move_ref_S(1,1)”

Done, thank you.

10 268

Correction “…being s…” should be “…where s is sampled…”

Done, thank you.

11 290

Correction “5.050” should be “5050”

Done, thank you.

12 310-312

Run-on sentence. The phrasing of this sentence is difficult to follow due to complex construction and use of commas.

Done, thank you.

13 337

Unclear. It is not clear what constitutes the head of the robot.

Done, thank you.

13 339

Introduction of shorthand text.

and should be introduced as shorthand for pitch and roll.

Done, thank you.

13 354/Eq. 8

Require further explanation. It is not obvious what the practical limit is or how it was determined.

Done, thank you.

14 383

Require further explanation. It is not obvious what and are in the equation.

Done, thank you.

18 448

Correction “once” should be “since”

Done, thank you.
