# Supplementary material for: Environmental influences on evolvable robots
Source: PLoS One. 2020 May 29;15(5):e0233848. doi: 10.1371/journal.pone.0233848 (PMC7259730; doi:10.1371/journal.pone.0233848)
Supplement: Attachment — Submitted filename: Response to Reviewers.pdf [file pone.0233848.s005.pdf]

Thank you very much for the review. My answers follow below in green. As for the public data, I’ve added it to the supplementary materials (S3 Datasets).

Introduction

We hypothesize that it has to do with the relative simplicity of these environments compared to the richness of (environmental) factors that determine the selection probabilities in Nature.

“in Nature” -> “in nature”.

I would suggest rephrasing to just say “selection in nature” as natural selection may well be a bit more complex than a set of probabilities.

Done: selection pressure in nature

“Curiously, in these (unpublished) experiments the same type of robots evolved in very different environments.”

I assume these unpublished experiments were those previously done by the authors? – If so I would recommend placing a footnote here – with a link to the results (ideally graphs) on an online repository – so as interested readers can look at them for reference and comparison.

I added it to the supplementary materials (S2 Appendix)

3.8 Mutation

“adding/deleting/swapping” -> adding, deleting or swapping

Done: adding, deleting or swapping

“production-rule/position” -> production-rule/position

Done: random production-rule in a random position

Also, fix in other sections: / should be replaced by “,” and “or” (as above).

Done.

Throughout the article, make sure there are no sentences split between pages – e.g.

“into two steps. The steps are illustrated by Fig 3.” On page 9.

Done.

Also, throughout the article, replace: “i.e.” -> “that is,” and “e.g.” -> “for example,”

Done.

Conclusion

“they face during their life.” -> they face during their lifetime.

Done.

“Having in mind that in the current experiments robots presented a degradation in the average”

->

“In the current experiments robots demonstrated a degradation in the average”

Done: Because in the current experiments robots demonstrated a degradation in the average...
